# Lumbosacral Soft Tissue Mass in a Newborn: A Clinical Case with a Difficult Diagnosis

**DOI:** 10.3389/fped.2017.00226

**Published:** 2017-10-23

**Authors:** Simone Ceratto, Maria Eleonora Basso, Francesco Savino

**Affiliations:** ^1^Postgraduation School of Pediatrics, University of Turin, Turin, Italy; ^2^Department of Pediatrics, Oncology Unit, Regina Margherita Children’s Hospital, AOU Città della Salute e della Scienza, Turin, Italy; ^3^Department of Pediatrics, Early Infancy Subintensive Care Unit, Regina Margherita Children’s Hospital, AOU Città della Salute e della Scienza, Turin, Italy

**Keywords:** soft-tissue lesions, neoplasms, newborn, lumbosacral region, pediatric oncology

## Abstract

Many types of dorsal neoplasm of early infancy are described in literature ranging from benign to aggressive. Some are more common while others quite unusual. Here, we describe a newborn with a lumbosacral soft tissue mass. Positivity of S-100 and vimentin was compatible with the neural cell line and the high proliferation rate of major activity cells (biopsy Ki67 20%) suggests an aggressive nature. An exclusively surgical approach was chosen and no clinical or radiological signs of recurrence have been observed after 2 years of follow-up. This case is atypical for location, histological pattern, radiological aspect, and clinical behavior. Diagnosis is hard to define and limited to a mesenchymal neoplasia with myxoid tracts. The described aspects raise concerns about clinical and therapeutic approach, classification, and radiological follow-up of sacral tissue masses in newborns.

## Introduction

We present a peculiar and still unclear case of lumbosacral mass in a newborn. In literature, some types of soft tissue neoplasm are described, starting from the most frequent (rhabdomyosarcoma, congenital fibrosarcoma, and infantile hemangiopericytoma) (1) to the most unusual (sacrococcygeal chordoma and mesenchymal chondrosarcoma) (2, 3), but the histological and clinical aspects of this case are not clearly linked to any of these neoplasms.

## Clinical Presentation

A newborn female was admitted to our hospital in her first month of life for a visible right lumbosacral paramedian mass. Pregnancy was uneventful except for non-insulin dependent gestational diabetes.

She was a term delivery, with birth weight at the 97° percentile and head circumference above the 97° percentile. Brain echography in the first week of life, revealed no pathological findings. Clinical and radiological initial evaluations and the following follow-up did not show other pathological findings, except for hip dysplasia treated with hip abduction braces in the first months of life.

## Diagnosis and Outcome

**Figure 1:**
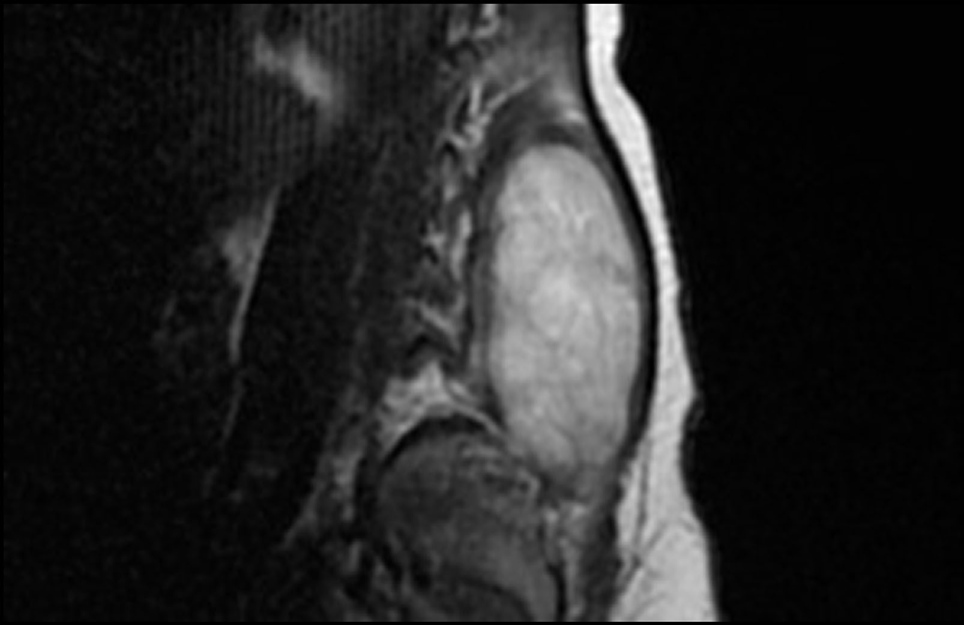
MRI before surgical excision (sagittal).

**Figure 2:**
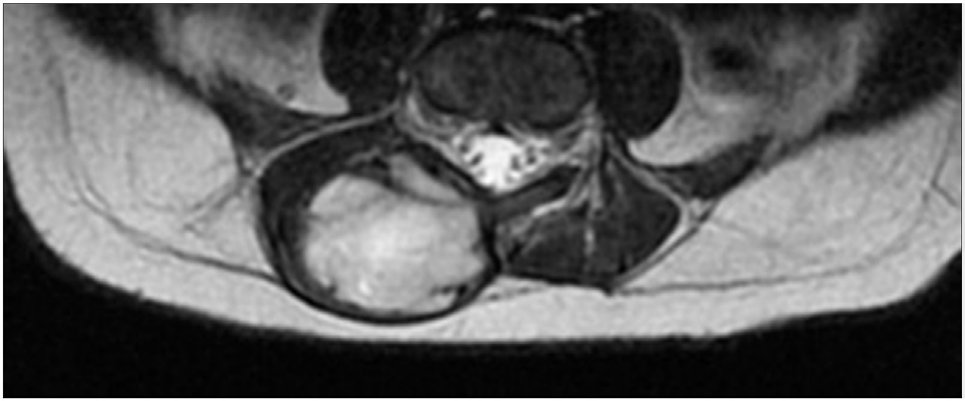
MRI before surgical excision (axial).

After admission, X-rays did not reveal clear bone involvement and an echography found an oval, strongly vascularized, lesion (36 × 15 mm), apparently connected to the spinal cord in the lumbar region. However, MRI (Figures 1 and 2) excluded spinal cord involvement and CT excluded bone involvement or erosions.

**Figure 3:**
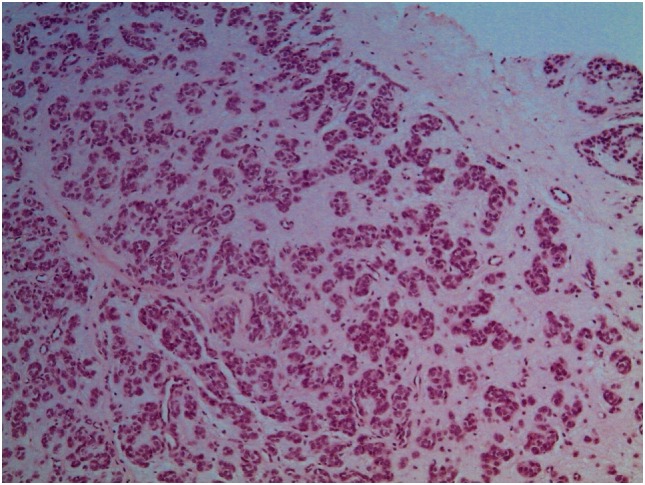
Biopsy.

A biopsy was performed following surgical and oncological evaluations (Figure 3).

A pathologist described the sample as a lesion formed by nests and tubules of medium size monomorphic cells with no cytological atypia. Immunohistochemical expression was compatible with the neural cell line (vimentin and S-100 positive; HUC/HUD, desmin, EMA, CD99, NSE, AE1-AE3, AFP, chromogranin, and GFAP negative). Proliferation index (Ki67) was 20% and only few mitoses were observed. Therefore, the mass seemed to be a non-aggressive soft tissues neoplasia with unidentified histotype.

Taking into account the child’s very young age and the histological characteristics of the lesion and considering that the macroscopic appearance suggested a good surgical resectability, a surgical approach was preferred to chemotherapy.

**Figure 4:**
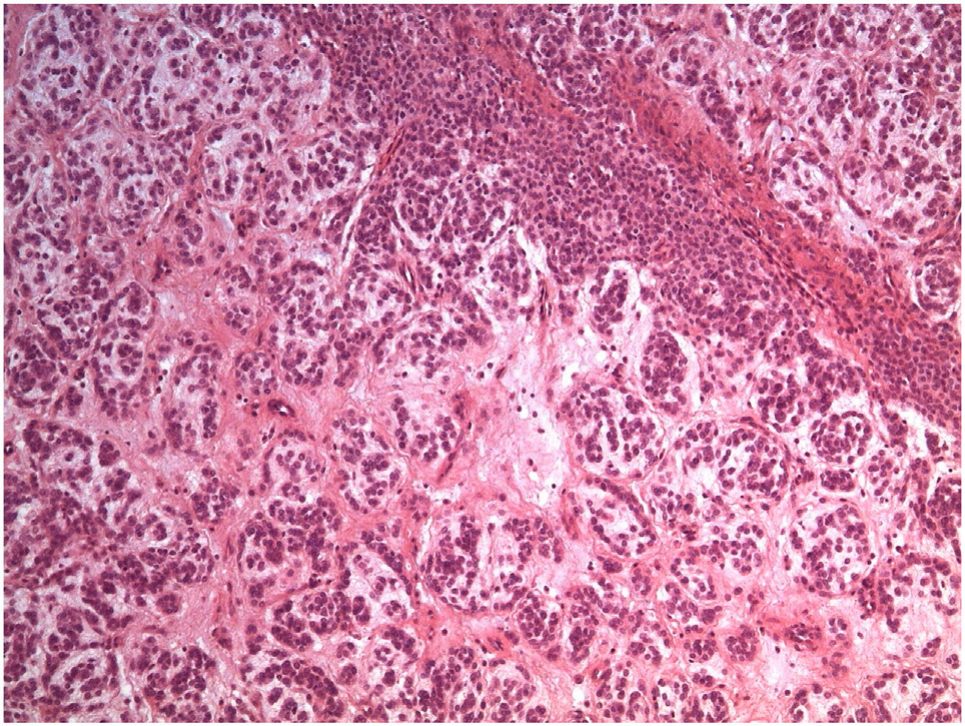
Removed mass.

Surgical excision of the mass was performed and then analyzed (Figure 4).

It was identified as a mesenchymal neoplasia, with myxoid tracts of uncertain classification, probably a chordoma (external to the central nervous system). Immunohistochemical expression resulted as follows: vimentin and S-100 positive; CD34 weak and focal positive; desmin, EMA, NSE, cytokeratin, inhibin, Melan A, synaptophysin, chromogranin, GFAP, estrogen, and progesterone receptors negative. Molecular analysis excluded rearrangements of EWSR1 and ETV6 genes and the presence of EWSR1/NR4A3 and TAF15/NR4A3 fusion transcripts.

Positivity of S-100 and the high proliferation rate (of major activity cells) also suggested a mesenchymal chondrosarcoma, but this possibility was excluded after further histological evaluation.

Further evaluations performed by Senior Pathologists from two other Centers (in Italy and USA) confirmed the uncertain classification of this neoplasm.

**Figure 5:**
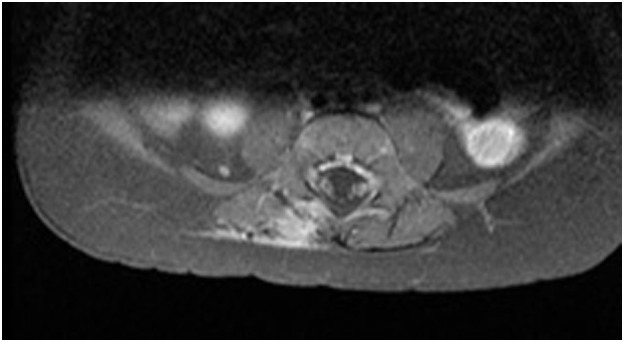
MRI after surgical excision (axial).

MRI performed 3 months later (Figure 5) found a small amount of tissue that was identified as post-surgical edema or scar tissue, although a neoplastic origin could not be excluded.

Three further MRI exams performed (each after 3 months) showed unvaried dimensions of the area and led to the second hypothesis. The fourth showed no pathological findings.

Up to now, the diagnosis is soft tissue sarcoma and a specific evaluation concerning prognosis is not possible, so the child is following a strict oncological follow-up.

## Discussion

The main types of soft tissue malignancy in infancy are rhabdomyosarcoma, congenital fibrosarcoma, and infantile hemangiopericytoma. Rhabdomyosarcoma is characterized by cellular necrosis and small round or large spindle cells (the latter usually has a better prognosis). Congenital fibrosarcoma shows a better prognosis than the adult type and is characterized by violet like skin discoloration and hypervascularity (that may lead to superficial ulcerations and disseminated intravascular coagulopathy). The lesion may cause bone deformity, but bone destruction is quite rare.

Microscopically, the lesion is highly cellular with spindle-shaped cells organized in fascicles or herringbone patterns and is frequently related to a specific translocation t(12;15).

Infantile hemangiopericytoma shows a more benign outcome than the adult type and is characterized by a hypervascularized soft tissue mass that sometimes causes bone invasion.

Peripheral nerve sheath neoplasia is also reported and is mainly associated to type 1 neurofibromatosis (1–4).

Transformation of a myxoid mesenchymal neoplasia of infancy to an undifferentiated sarcoma is reported in literature, so a long-term follow-up of these diseases is recommended (5).

Nonteratomatous neoplasms located in the sacral region in children form a very heterogeneous group without a prevalent type. Diagnosis is often late, but complete surgical resection is associated with a good prognosis (6).

Chordomas are nonteratomatous neoplasms, infrequent in infancy, and mainly localized in the cranial region. They are low-grade neoplastic lesions developing from notochordal residues. Sacral chordoma usually shows a worse prognosis due to the venous drainage density of the region.

Surgical resection and radiotherapy are the common treatments, but recently also photon therapy has been suggested (2).

Mesenchymal chondrosarcoma is a rare malignancy more frequent in young adults and prone to cause lung metastases. Surgical resection is the main treatment, while the efficacy of chemo and radiotherapy is not clear. Dantonello et al. reported that clinical and prognostic criteria of adults could not be extended to the pediatric population and suggest radiotherapy if complete surgical resection is not possible. They also proposed considering induction chemotherapy (3).

Habrand et al. reported the case of a 9-year-old boy with a chordoma of the cranial-cervical junction, treated with surgical resection, radiotherapy, and photon therapy. Ten years after treatment, MRI revealed a local tumor progression, treated with subtotal resection and radiotherapy. A year later, the patient died because of a hemorrhage that could have been caused by tumor progression or radionecrosis of the internal carotid artery (2).

Shinmura et al. reported the clinical case of a 3-year-old boy with a sacrococcygeal chordoma. When diagnosis was performed, it had infiltrated the bone and lung metastases were detectable on CT. Treatment was performed with chemo and radiotherapy, with little response. The child died 14 months after the first admission to hospital because of respiratory failure due to lung metastasis (7).

Namini et al. reported the case of a congenital sacrum mesenchymal chondrosarcoma in a newborn. The neoplasia was surgically removed, but 10 days later many metastases on the back were noted. Chemotherapy was suggested, but the parents refused. The infant died 3 months later (8).

Dorso-lumbar teratoma is a quite common location in early infancy, but this type of neoplasm usually shows a more typical histological pattern, characterized by mature and benign components, derived from all three germ layers. In infants, it is frequently associated with spinal dysraphisms or cord malformations. Radiologically, it shows mixed solid and cystic morphology, fat signals, and calcifications (9).

A case of soft-tissue sarcoma associated with Sotos syndrome has been described by Hill et al. (10), but our patient, despite neonatal weight and head circumference, did not show any clinical or radiological characteristics of this syndrome, so this hypothesis can not be taken into account.

## Conclusion

The described clinical case is peculiar for two reasons: first, the histological aspect of the mass does not allow a clear identification of the neoplasm and so a prognostic evaluation cannot be performed. Second, according to literature, soft tissue malignancies presenting a similar macroscopic aspect and localization in children, usually are more aggressive and invasive than in this case. Because of these uncertainties, a strict follow-up was strongly recommended. The child’s age raises some concerns: muscular tissue is still growing, so a more accurate histological evaluation is difficult. Furthermore, the child must be protected from side effects due to follow-up exams (e.g., narcosis for MRI). At the same time, the distinction between scar and neoplastic tissue is mandatory to avoid recurrences.

Taking into account the favorable behavior of the lesion (compared to similar neoplasms presented in literature) and the unclear diagnosis (as confirmed by experts from two external centers), we decided to describe this case and to share our findings with other physicians involved in neonatal and child care, with the hope that this will lead to further progress in the field.

Managing lumbosacral soft tissue masses in newborns and infants is a challenge involving many health professionals (neonatologists, pediatricians, oncologists, surgeons, and radiologists) because of the large range of possible diagnoses and the lack of standardized approaches.

## Ethics Statement

Patient’s parents approved the publication with a written consent form.

## Author Contributions

SC and FS wrote the paper. MB revised the paper.

## Conflict of Interest Statement

The authors declare that the research was conducted in the absence of any commercial or financial relationships that could be construed as a potential conflict of interest.
